# Expression of Mitochondrial Regulators PGC1α and TFAM as Putative Markers of Subtype and Chemoresistance in Epithelial Ovarian Carcinoma

**DOI:** 10.1371/journal.pone.0107109

**Published:** 2014-09-22

**Authors:** Marike Gabrielson, My Björklund, Joseph Carlson, Maria Shoshan

**Affiliations:** 1 Department of Oncology-Pathology, Cancer Center Karolinska CCK, Karolinska Institutet, Stockholm, Sweden; 2 Department of Pathology-Cytology, Radiumhemmet, Karolinska University Hospital, Stockholm, Sweden; Florida International University, United States of America

## Abstract

Epithelial ovarian carcinoma (EOC), the major cause of gynaecological cancer death, is a heterogeneous disease classified into five subtypes. Each subtype has distinct clinical characteristics and is associated with different genetic risk factors and molecular events, but all are treated with surgery and platinum/taxane regimes. Tumour progression and chemoresistance is generally associated with major metabolic alterations, notably altered mitochondrial function(s). Here, we report for the first time that the expression of the mitochondrial regulators PGC1α and TFAM varies between EOC subtypes; furthermore, we have identified a profile in clear-cell carcinoma consisting of undetectability of PGC1α/TFAM, and low ERα/Ki-67. By contrast, high-grade serous carcinomas were characterised by a converse state of PGC1α/TFAM, ERα positivity and a high Ki-67 index. Interestingly, loss of PGC1α/TFAM and ERα was found also in a non-clear cell EOC cell line made highly resistant to platinum in vitro. Similar to clear-cell carcinomas, these resistant cells also showed accumulation of glycogen. Altogether, our data provide mechanistic insights into the chemoresistant nature of ovarian clear-cell carcinomas. Furthermore, these findings corroborate the need to take into account the diversity of EOC and to develop subtype specific treatment strategies.

## Introduction

Epithelial ovarian carcinoma (EOC) is the major cause of gynaecological cancer death in women [1]. It is treated with surgery and platinum/taxane regimens as first-line therapy, but despite initial responsiveness, >75% of patients relapse into metastatic and essentially incurable chemoresistant disease [1], [2]. EOC is a heterogeneous disease that is broadly classified as serous (high- and low-grade serous carcinoma (HGSC and LGSC, respectively)), or non-serous (endometrioid, EC; mucinous, MC; clear-cell, CCC) histology. Each subtype has distinct clinical characteristics and is associated with different genetic risk factors and molecular events [3], [4]. Sensitivity to chemotherapy also varies between them, and survival correlates with subtype [5]. In CCC, both response rates and five-year survival are lower than in HGSC [5]–[7] and CCC has been generally accepted as an aggressive subtype that is poorly responsive to existing chemotherapy regimes.

To support the increased demand for energy and building blocks during tumour progression, cancer cells undergo major metabolic alterations [8], [9]. Chemoresistance and metastasis are associated with such alterations, notably altered mitochondrial function(s) [8]. Whereas normal cells depend on mitochondrial oxidative phosphorylation (OxPhos) for energy, tumour cell metabolism includes, among other pathways, decreased OxPhos and increased glycolysis (the Warburg effect) and glutaminolysis, both of which require mitochondria [9], [10]. Tumour cells also show survival-promoting alterations e.g. in regulation of autophagy in response to nutritional and other conditions of cellular/metabolic stress; these processes involve the AMP-activated protein kinase AMPK [11].

In normal cells, the transcriptional coactivator peroxisome proliferator-activated receptor gamma coactivator 1-alpha (PGC1α) is a major coordinator of metabolism and mitochondrial function [12] and together with SIRT1 and AMPK constitutes a sensitive network responding to metabolic stress [13]. Together with SIRT1, PGC1α has been reported to reside inside mitochondria with direct functions on mitochondrial biofunction [14]. PGC1α also regulates mitochondrial biogenesis through regulation of NRF1-2 [15], which in turn regulates mitochondrial transcription factor A (TFAM) [16]. TFAM plays a critical role in maintaining copy number and structure of mtDNA [17], [18], and is hence crucial for efficient transcription of mtDNA genes such as cytochrome c oxidase subunit 2 (MT-CO2) and other OxPhos proteins [17]. TFAM binding to mtDNA is regulated via phosphorylation [19]; unbound TFAM may be degraded by the Lon protease [19], [20]. Although well studied in normal cells, for instance regarding aging and neurodegeneration [21], [22], diabetes and obesity [23], it remains unknown what roles PGC1α/TFAM play in tumour progression.

Mitochondrial biogenesis has, in addition to PGC1α, in several studies been linked to expression and localisation of oestrogen (E_2_) and oestrogen receptor alpha (ERα) [24], [25]. The EOC subtypes express ERα at different levels, and expression is lowest in CCC [26].

The aim of this study was to examine the role of mitochondrial regulation in EOC by examining and correlating the expression of the mitochondrial and metabolic markers PGC1α, TFAM and ERα in clinical samples, and to relate the expression to EOC subtypes. Furthermore, we examined these markers in an EOC cell line and a multiresistant subline thereof.

## Materials and Methods

### Cells and cell culture

The non-CCC EOC cell line SKOV-3 and a multiresistant subline, SKOV-3-R (previously described in [27]) were cultured at +37°C, in 5% CO_2_ in RPMI-1640 medium containing 2 mM L-glutamine, 5% fetal bovine serum and 1% penicillin–streptomycin (all from Nordic Biolabs). SKOV-3 identity was validated using short tandem repeat analysis AmpF*l*STR Identifiler kit (Applied Biosystems/Life Technologies) and cultures were routinely tested for mycoplasma contamination.

### Quantitative real-time PCR

RNA was extracted using Qiagen RNeasy mini kit (Qiagen) and 500 ng/sample was used for the generation of cDNA using High Capacity cDNA Reverse Transcription Kit (Life Technologies) according to manufacturer's instructions. Quantitative real-time PCR (qRT-PCR) was performed on a 7900 HT Fast Real-Time system using TaqMan Gene expression assays (PPARC1A: Hs01016719_m1, TFAM: Hs01082775_m1, ESR1: Hs00174860_m1) and TaqMan Fast Universal PCR Master Mix (all from Applied Biosystems/Life Technologies) with the following cycling conditions: 95°C for 20 sec, 40 cycles of 95°C for 1 sec and 60°C for 20 sec. Expression of ACTB (Hs99999903_m1) was used as an endogenous control and the fold gene expression was calculated using the 2^−ΔΔCT^-method [28]. Raw data are shown in file Raw Data S1.

### Western blot

Cells were lysed in RIPA lysis buffer (1% NP-40, 0.5% sodium deoxycholate, 0.1% sodium dodecyl sulphate, 0.004% sodium azide) containing protease and phosphatase inhibitor cocktails (all from Sigma-Aldrich). Samples were loaded for gel electrophoresis at 20 µg/sample and blotted onto PVDF membranes. Primary rabbit antibodies were to: PGC1α, β-tubulin (both from Abcam, 1∶1,000, 1∶5,000 respectively) and TFAM (1∶1,000, Sigma-Aldrich). Primary mouse antibodies were to ERα (1∶1,000, Santa Cruz Biotechnology) and Ki-67 (1∶500, Dako). Secondary HRP-conjugated antibodies used were donkey anti-rabbit IgG (1∶2,500, Abcam) and sheep anti-mouse IgG (1∶5,000, GE Healthcare). Images were developed with Western Lightning Plus-ECL (PerkinElmer) and captured using Protec Optimax X-Ray film processor.

### Study population and ethical considerations

The study set (n = 53 cases) was drawn from cases originally diagnosed at the Karolinska University Hospital (Stockholm, Sweden). Participants provided written informed consent and the study was carried out with the approval of the Regional Ethics Committee of Stockholm (EPN 2013/318-31/2). Two pathologists reviewed all cases. The diagnoses included in the study set included 21 HGSC (40%), 14 CCC (26%), ten EC (19%), 7 MC (13%) and one LGSC (2%). Clinicopathological features of the study population can be found in Table 1. A tissue microarray (TMA) was constructed using paired 1 mm cores from representative tumour material. Representativity was confirmed using a hematoxylin and eosin control section.

**Table 1:** Study population.

Clinical Variable	All	High-grade serous	Clear cell	Endometrioid	Mucinous	Low-grade serous	*p*-value^a^
Number of cases, n (%)	53 (100)	21 (40)	14 (26)	10 (19)	7 (13)	1 (2)	
Age in years, median (range)	64.0 (25–90)	66.0 (36–83)	63.5 (40–87)	55.5 (43–88)	64.0 (25–90)	n.d.	n.s.
Ki-67, median % (range)	31.2 (1–90)	38.8 (12–90)	20.2 (1–46)	19.3 (1–43)	31.2 (12–49)	n.d.	0.003
ERα							<0.001
Negative, n (%)	23 (43)	2 (10)	12 (86)	2 (20)	7 (100)	0 (0)	
Positive, n (%)	30 (57)	19 (90)	2 (14)	8 (80)	0 (0)	1 (100)	
PR							0.038
Negative, n (%)	36 (68)	14 (67)	11 (79)	4 (40)	7 (100)	0 (0)	
Positive, n (%)	17 (32)	7 (33)	3 (21)	6 (60)	0 (0)	1 (100)	
Histological Stage							0.001
I	22 (42)	2 (10)	7 (50)	8 (80)	5 (71)	0 (0)	
II	5 (9)	2 (10)	2 (14)	1 (10)	0 (0)	0 (0)	
III	26 (49)	17 (81)	5 (36)	1 (10)	2 (29)	1 (100)	

Abbreviations: ERα; oestrogen receptor alpha, nd; not determined, ns; not significant, PR; progesterone receptor.

a Across variable; tested with Fisher's exact test for subtype, expression of ERα and PR, and Kruskal-Wallis test for distribution of age and Ki-67.

### Immunohistochemistry and antibodies

Immunohistochemical staining (IHC) was performed on whole TMA using the EnVision + System (Dako). Antigen retrieval was achieved by immersing the sections in citrate buffer at pH 6.0 and boiling for 20 minutes in a water bath. The sections were then incubated with primary rabbit polyclonal antibodies against human voltage-dependent anion channel (VDAC) and PGC1α (both from Abcam, 1∶800 and 1∶100, respectively), TFAM and Lon (both from Sigma-Aldrich, 1∶10 and 1∶800, respectively) and rabbit monoclonal antibodies against human MT-CO2 (1∶100, Abcam) for 30 min at room temperature. A positive reaction was detected using the EnVision +System (Dako) and slides were counterstained with hematoxylin (Dako). Staining of Ki-67, ERα and progesterone receptor (PR) was performed at the routine laboratory at the Department of Pathology-Cytology, Karolinska University Hospital (Stockholm, Sweden), using rabbit monoclonal antibodies from Ventana Medical Systems.

### Evaluation of immunohistochemistry

For evaluation of immunoreactivity and histological appearance, all TMA slides were examined independently by two investigators (MG and MS). Positive cytoplasmic expression of VDAC, ERα, PGC1α, TFAM, MT-CO2 and Lon was defined as immunoreactivity in >1% of epithelial cells, while immunoreactivity in <1% of epithelial cells defined negative protein expression. The scoring for each individual sample can be seen in the Raw Data S1. Ki-67 index was assessed as a continuous variable as percentage of positive tumour cells.

### Glycogen assay

Cells were grown to confluency prior to harvesting. Intracellular levels of glycogen were then detected in 1×10^6^ cells using the colorimetric Glycogen Assay Kit II (Abcam) according to manufacturer's instructions.

### Sulforhodamine B assay

Growth rates in SKOV-3 and SKOV-3-R cells were evaluated based on total cellular protein in the samples, in turn assessed using the sulforhodamine B-based TOX6 kit (SRB assay, Sigma-Aldrich) according to manufacturer's instructions. Briefly, SKOV-3 and SKOV-3-R cells were seeded in 96-well plates at 4×10^3^ cells/well in quadruplicates for each time point. After overnight incubation the wells for *t* = 0 h were fixed while the remaining wells were incubated additional 72 h before fixation. Resulting absorbances (i.e. total cellular protein) were compared with that at *t* = 0 h.

### siRNA transfection

SKOV-3 cells seeded at 7.5×10^4^ cells/well (12-well plates) or 15×10^4^ cells/well (6-well plates) were transfected with 50 nM Silencer siRNA specific for PPARGC1A or with 50 nM Silencer Negative Control No. 1 siRNA (Life Technologies, id: 114747 and AM4611, respectively) after overnight incubation. siRNA, diluted in Opti-MEM (Life Technologies), was mixed by vortexing with 3 µl HiPerFect Transfection Reagent (Qiagen) per 100 µl Opti-MEM/siRNA mixture followed by 10 min incubation at RT. Subsequently, transfection complexes were added to the cells and incubated for 5 h under normal growth conditions, whereafter these complexes were replaced by antibiotic-free growth medium. At 72 h post-transfection, mRNA and protein levels were analysed by qRT-PCR and western blot, respectively. In parallel experiments, at 24 h post-transfected cells were treated with cisplatin (Hospira) for 48 h. Response to treatment was evaluated using the SRB assay.

### Statistical analysis

To assess the relationship between protein expression levels and the variables in the clinical material, a non-parametric Chi^2^ test or Fisher's exact test was used as indicated in the tables. Comparisons between groups were assessed using the independent t-test (*in vitro* data), Mann-Whitney U test or Kruskal-Wallis test as indicated (IHC data). A two-tailed P value <0.05 was considered significant, and *p* values <0.05, <0.01 or <0.001 are represented with one, two or three asterisks, respectively, in the figures. IBM SPSS 20.0 statistical software (SPSS Inc.) was used for all analyses. For IHC analysis of MT-CO2 and Lon, two and five samples, respectively, were unavailable for scoring, which was taken into account in the statistical analysis.

## Results

### PGC1α and TFAM expression in multiresistant EOC cells

**Figure 1:**
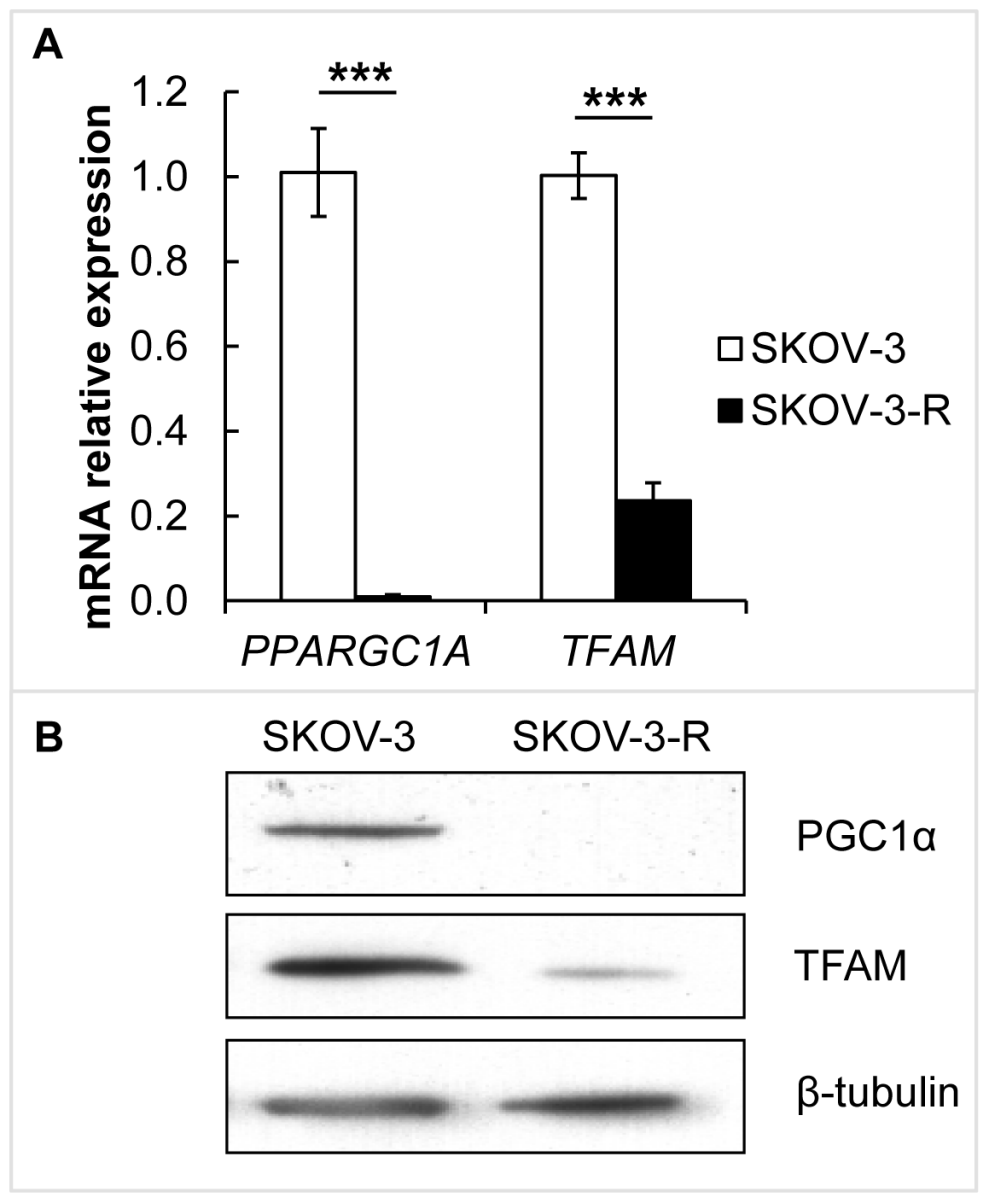
PGC1α and TFAM expression in EOC SKOV-3 cells and the multiresistant subline SKOV-3-R. (A) Gene expression of *PPARGC1A* and *TFAM* in SKOV-3 and SKOV-3-R cells was evaluated by qRT-PCR (*n* = 3). The decrease in expression in SKOV-3-R was statistically significant (both *p*<0.001, using independent t-test). Expression levels were normalised to *ACTB*. Error bars represent S.E.M. (B) Representative western blot showing protein expression of PGC1α and TFAM. β-tubulin was used as loading control.

The EOC cell line SKOV-3 was subjected to repeated long-term treatment with cisplatin generating the multiresistant subline SKOV-3-R, as previously described in [27]. Since mitochondrial content was previously found to be higher in the resistant subline [27], expression of mitochondrial regulators PGC1α and TFAM was investigated here. mRNA expression of the genes coding for PGC1α and TFAM (*PPARGC1A* and *TFAM*, respectively) was significantly decreased in SKOV-3-R compared to parental SKOV-3 (*p*<0.001 for both) (Fig. 1A). Accordingly, protein expression of PGC1α and TFAM was reduced in SKOV-3-R cells (Fig. 1B).

### Clinicopathological features of the cohort

Immunohistochemistry was used to analyse the expression of selected proteins in 53 cases of EOC, including 21 HGSC (40%), 14 CCC (26%), ten EC (19%), seven MC (13%) and one LGSC (2%). Patient and tumour characteristics are described in Table 1. Representative images of IHC staining for Ki-67, MT-CO2 and Lon are shown in Fig. S1.

No significant difference in median age was observed between the subtypes. Significant differences between subtypes were found for the biomarkers Ki-67 (*p* = 0.003) Figs. S1–S2), ERα (*p*<0.001), PR, (*p* = 0.038) as well as for the distribution across histological stages (*p* = 0.001) (Table 1). Within HGSC, 19 tumours (90%) were positive and 2 (10%) were negative for ERα (*p*<0.001), whereas in CCC 2 tumours (14%) were positive and 12 (86%) were negative (*p* = 0.008).

The mitochondrial outer membrane VDAC was used as a positive control for presence of mitochondria. Expression of VDAC was found in all samples and was equal across all EOC subtypes (not shown).

### Expression of PGC1α and TFAM in EOC tumours

Protein expression of PGC1α was cytoplasmic and seen in 42 tumours (79%); conversely, eleven tumours (21%) lacked PGC1α. The expression varied significantly across all EOC subtypes (*p* = 0.002). In HGSC, 18 tumours (86%) were positive and three (14%) were negative for PGC1α (*p*<0.001). In CCC, six tumours (43%) were positive whereas eight (57%) were negative. All EC, MC and LGSC tumours were positive for PGC1α. In the total cohort, there was no significant correlation between PGC1α, Ki-67 index, PR, age or histological stage. By contrast, there was a significant positive correlation between ERα and PGC1α (*p*<0.001) (Table 2).

**Table 2:** Expression status of PGC1α and TFAM in the EOC study population.

	PGC1α				TFAM			
Clinical Variable	Negative	Positive	*p*-value^a^	*p*-value^b^	Negative	Positive	*p*-value^a^	*p*-value^b^
Number of cases, n (%)	11 (21)	42 (79)			18 (34)	35 (66)		
Subtype			0.002				0.001	
High-grade serous, n (%)	3 (14)	18 (86)		0.001	3 (14)	18 (86)		0.001
Clear cell, n (%)	8 (57)	6 (43)		n.s.	11 (79)	3 (21)		0.033
Endometrioid, n (%)	0 (0)	10 (100)		n.d.	3 (30)	7 (70)		n.s.
Mucinous, n (%)	0 (0)	7 (100)		n.d.	1 (34)	6 (66)		n.s.
Low-grade serous, n (%)	0 (0)	1 (100)		n.d.	0 (0)	1 (100)		n.d.
Age in years, median (range)	61.0 (40–87)	64.5 (25–90)	n.s.		65.5 (40–87)	63.0 (25–90)	ns	
Ki-67, median % (range)	32.5 (1–52)	30.0 (1–90)	n.s.		20.2 (1–68)	33.2 (1–90)	0.014	
ERα			<0.001				0.014	
Negative, n (%)	10 (44)	13 (57)			12 (52)	11 (48)		
Positive, n (%)	1 (3)	29 (97)			6 (20)	24 (80)		
PR			n.s.				n.s.	
Negative, n (%)	10 (28)	26 (72)			15 (41)	21 (58)		
Positive, n (%)	1 (6)	16 (94)			3 (18)	14 (82)		

Abbreviations: EOC; epithelial ovarian carcinoma, ERα; oestrogen receptor alpha, nd; not determined, ns; not significant, PR; progesterone receptor, PGC1α; Peroxisome proliferator-activated receptor gamma co-activator 1-alpha, TFAM; mitochondrial transcription factor A.

a Across variable; tested with Fisher's exact test for subtypes, expression of ERα and PR, and Mann-Whitney U test for distribution of age and Ki-67.

b Within variable; tested with non-parametric Chi^2^ test.

Expression of TFAM was cytoplasmic, and varied significantly across all EOC subtypes (*p* = 0.001). In total, TFAM was expressed in 35 tumours (66%) and undetectable in 18 tumours (34%). In HGSC, 18 tumours were positive and three were negative for TFAM (86% and 14%, respectively) (*p* = 0.001). In CCC, three tumours were positive whereas eleven were negative (21% and 79%, respectively) (*p* = 0.033). In EC, seven tumours were positive and three were negative (70% and 30%, respectively). In MC, six tumours were positive and one was negative (66% and 34%, respectively), and in LGSC, the only tumour included expressed TFAM (100%).

**Figure 2:**
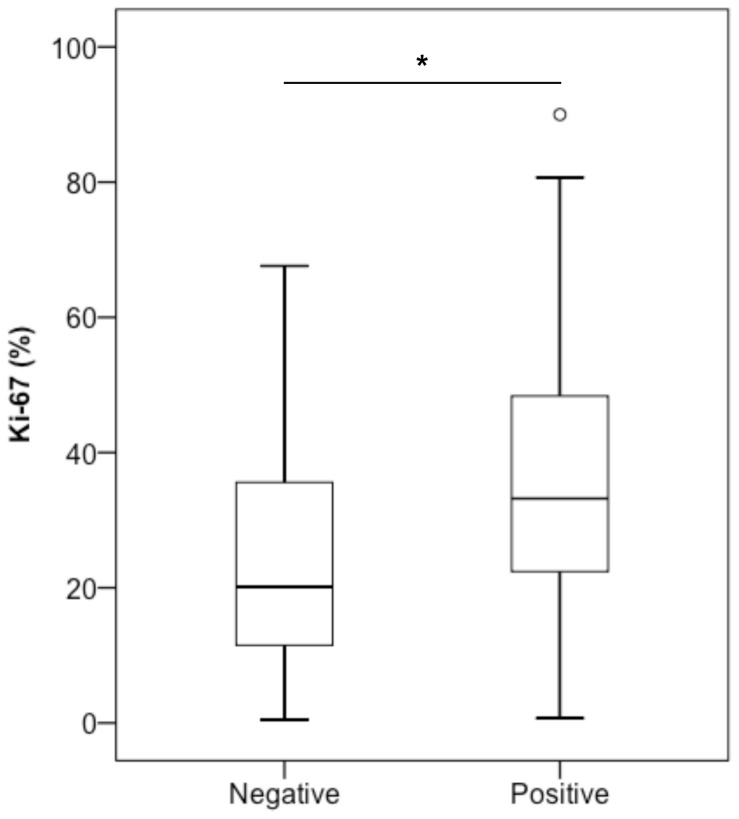
Ki-67 index distribution in TFAM+ and TFAM- EOC tumours. EOC tumours with negative expression of TFAM (*n* = 18) showed significantly lower Ki-67 index compared to tumours with positive expression of TFAM (*n* = 35) (Mann-Whitney U, *p* = 0.014).

Across the EOC subtypes, there was no significant correlation between TFAM, PR, age or histological stage. However, tumours expressing TFAM displayed a significantly higher Ki-67 index compared to TFAM negative tumours (median score 33% and 20%, respectively) (*p* = 0.014) (Fig. 2). There was also a significant positive correlation between TFAM and expression of ERα (*p* = 0.014). In order to investigate if lack of TFAM expression affected the expression of mtDNA-encoded genes, protein expression of MT-CO2 was assessed. Of 51 tumours stained for MT-CO2, 47 (92%) were positive and four (8%) were negative. Three of the four MT-CO2 negative tumours were also TFAM negative; however, there was no significant relation between expression of MT-CO2 and TFAM (Table S1 in File S1).

TFAM protein levels are in part regulated through degradation by the Lon protease [19], [20]. To investigate if increased levels of Lon might explain the reduced levels of TFAM found in CCC, expression of Lon was assessed. Lon was localised to the cytoplasm, and was found in 38 tumours (79%) which were differently distributed across all EOC subtypes (*p* = 0.032). There was no significant correlation between expression of TFAM and Lon (Table S2 in File S1).

### Co-expression of PGC1α and TFAM in EOC tumours

**Figure 3:**
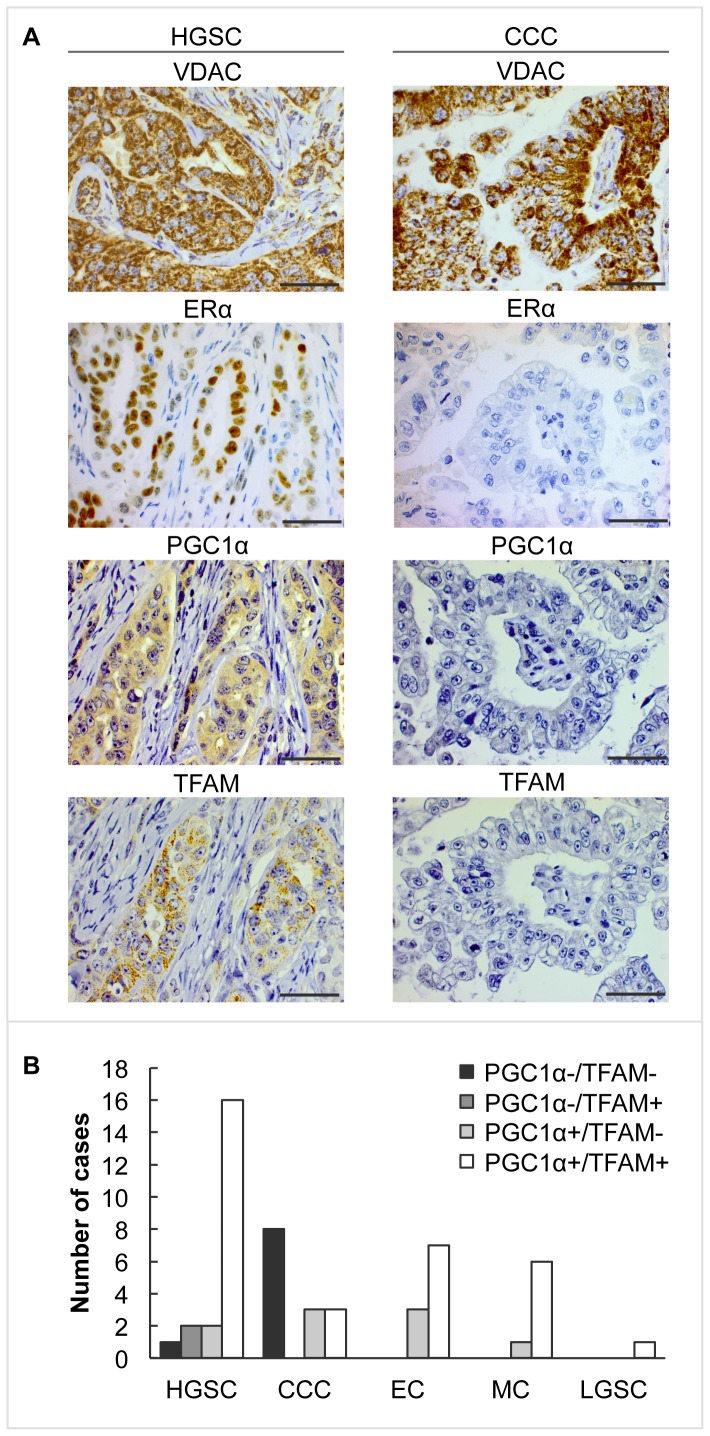
Expression of PGC1α and TFAM in EOC subtypes. (A) Representative immunohistochemical staining of VDAC, ERα, PGC1α and TFAM in HGSC and CCC. VDAC indicates presence of mitochondria. ERα, PGC1α and TFAM were all expressed in HGSC, whereas in CCC expression of the proteins was lost. Each HGSC and CCC quadruplicate is from one and the same patient (magnification: 400x, scale bar shows 500 µm). (B) Histogram representing number of cases with expression of PGC1α/TFAM across EOC subtypes.

As PGC1α is a major coordinator of mitochondrial functions including regulation of transcription of TFAM [12], [15], [16], we investigated the co-expression of these two proteins. The cohort was categorised into four different groups depending on their expression of PGC1α and TFAM (PGC1α-/TFAM-; PGC1α-/TFAM+; PGC1α+/TFAM- and PGC1α+/TFAM+). The expression of PGC1α/TFAM varied significantly across the EOC subtypes (*p* = 0.001) (Table 3, Fig. 3). There was also a significant difference in median Ki-67 index between the four groups, where positive expression of TFAM associated with increased Ki-67 index independently of PGC1α expression (*p* = 0.048) (Table 3, and Fig. S3). Furthermore, there was a positive association between ERα expression and co-expression of PGC1α/TFAM (*p* = 0.002) (Table 3), whereas no significant association with PR was observed.

**Table 3:** Expression status of PGC1α/TFAM in EOC.

Clinical Variable	PGC1α-/TFAM-	PGC1α-/TFAM+	PGC1α/TFAM-	PGC1α/TFAM+	*p*-value^a^
Number of cases, *n* (%)	9 (17)	2 (4)	9 (17)	33 (62)	
Subtype					0.001
High-grade serous, *n* (%)	1 (5)	2 (10)	2 (10)	16 (76)	
Clear cell, *n* (%)	8 (57)	0 (0)	3 (21)	3 (21)	
Endometrioid, *n* (%)	0 (0)	0 (0)	3 (30)	7 (70)	
Mucinous, *n* (%)	0 (0)	0 (0)	1 (14)	6 (86)	
Low-grade serous, *n* (%)	0 (0)	0 (0)	0 (0)	1 (100)	
Age in years, median (range)	61.0 (40–87)	55.0 (44–66)	70.0 (53–80)	63.0 (25–90)	n.s
Ki-67, median % (range)	24.7 (1–43)	49.5 (48–52)	17.9 (5–68)	32.8 (1–90)	0.048
ERα					0.002
Negative, *n* (%)	8 (35)	2 (9)	4 (17)	9 (39)	
Positive, *n* (%)	1 (3)	0 (0)	5 (17)	24 (80)	
PR					n.s.
Negative, *n* (%)	8 (22)	2 (6)	7 (19)	19 (53)	
Positive, *n* (%)	1 (6)	0 (0)	2 (12)	14 (82)	

Abbreviations: EOC; epithelial ovarian carcinoma, ERα; oestrogen receptor alpha, nd; not determined, ns; not significant, PR; progesterone receptor, PGC1α; Peroxisome proliferator-activated receptor gamma co-activator 1-alpha, TFAM; mitochondrial transcription factor A.

a Across variable; tested with Fisher's exact test for subtype, expression of ERα and PR, and Kruskal-Wallis test for distribution of age and Ki-67.

Co-expression of PGC1α/TFAM was found in 33 samples (62% of the total cohort) of which 16 (48%) were HGSC. Within the HGSC subtype, 76% of the tumours were double-positive for PGC1α/TFAM (*p*<0.001). Lack of both PGCα and TFAM was observed in nine tumours (17% of total cohort). Of these nine, all but one were CCC (89% of all double negative) (*p* = 0.020). Within the CCC subtype, double-negative expression occurred in 57% of the tumours and all of these were ERα negative (Table 3).

### CCC profile of multiresistant EOC cell line

**Figure 4:**
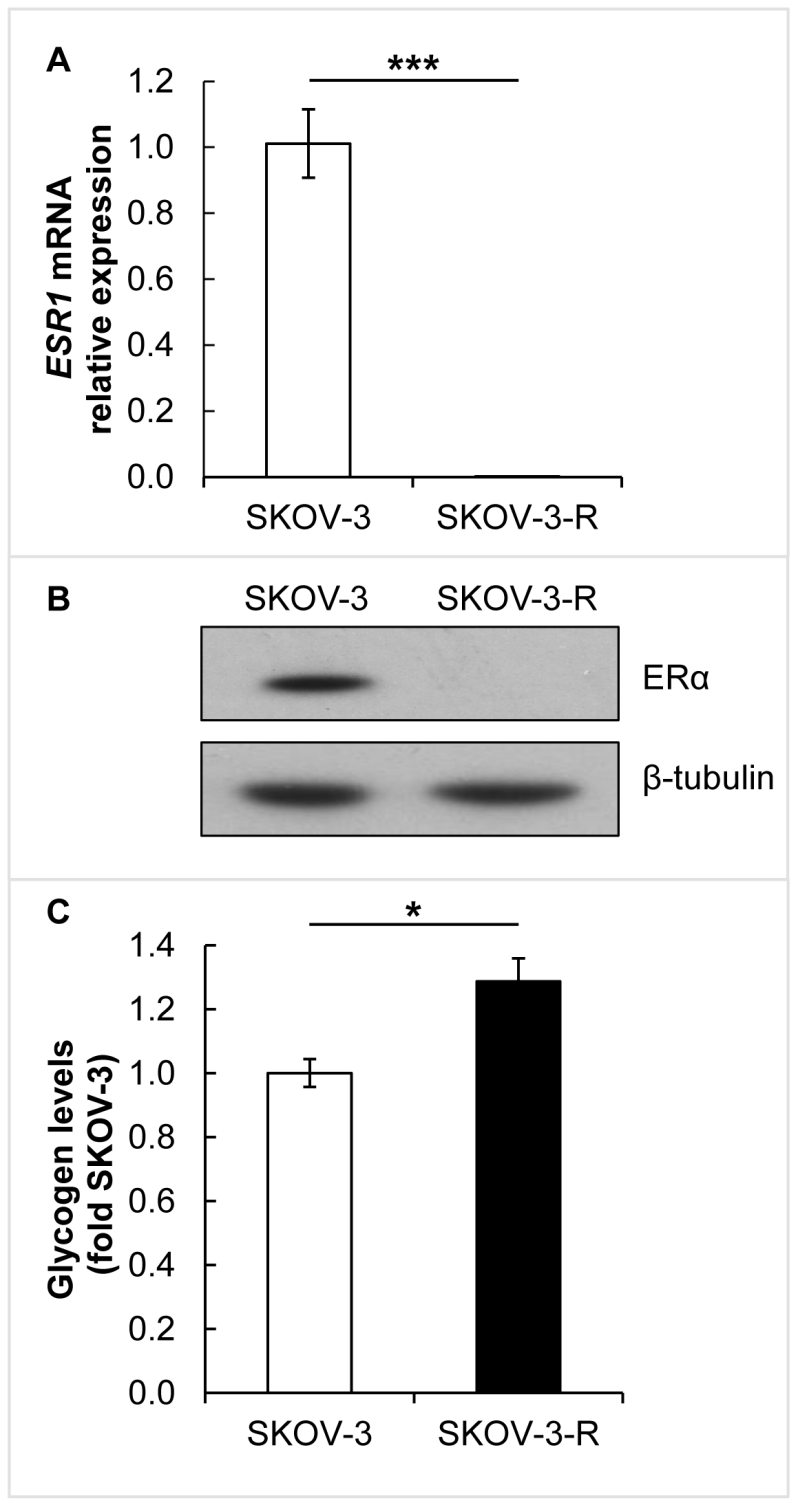
ERα expression and intracellular levels of glycogen in SKOV-3 and SKOV-3-R. (A) Gene expression of *ESR1* evaluated by qRT-PCR (*n* = 3). The decrease in expression in SKOV-3-R was statistically significant (*p*<0.001, using independent t-test). Expression levels were normalised to *ACTB*. Error bars represent S.E.M. (B) Representative western blot showing protein expression of ERα. β-tubulin was used as a loading control. (C) Glycogen levels were evaluated using a colorimetric assay (*n* = 3). The increased level in SKOV-3-R was statistically significant (*p* = 0.027, using independent t-test). Error bars represent S.E.M.

SKOV-3 cells have been reported to have a 32-basepair deletion in exon 1 of ERα transcript and are thus ERα positive but oestrogen-insensitive [29], [30]. To further investigate the link between regulation of ERα expression, as such, and PGC1α/TFAM, levels of ERα were analysed on mRNA and protein levels. Compared to parental SKOV-3, the multiresistant SKOV-3-R cells show significantly decreased mRNA expression of the *ESR1* gene (*p*<0.001) (Fig. 4A), and accordingly decreased ERα protein expression (Fig. 4B). Raw data can be found under S_PCR in the file Raw data S1s.

Accumulation of glycogen is characteristic of CCC [31], wherefore we investigated intracellular glycogen levels in SKOV-3 and SKOV-3-R cells. The SKOV-3-R were found to accumulate 25% more intracellular glycogen (*p* = 0.027) compared to parental cells (Fig. 4C).

## Discussion

**Figure 5:**
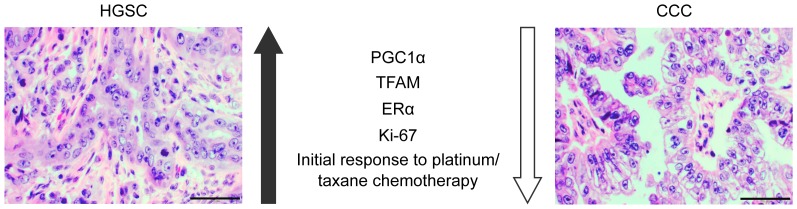
Comparison of ovarian HGSC and CCC. Representative hematoxylin and eosin staining of ovarian HGSC and CCC. We hypothesise that these subtypes represents extreme forms of EOC; HGSC generally display positive expression of PGC1α, TFAM and ERα, have a higher Ki-67 index and are more responsive to initial platinum/taxane chemotherapy. By contrast, CCC generally do not express PGC1α, TFAM and ERα, have a lower Ki-67 index and are less responsive to initial platinum/taxane chemotherapy. Magnification 400x, scale bar shows 50 µm.

We have here shown that the expression of mitochondrial regulators PGC1α and TFAM varies significantly between HGSC and CCC. Furthermore, we identified a profile in CCC consisting of undetectability of PGC1α/TFAM, and low ERα/Ki-67. By contrast, HGSC samples were characterised by a converse state of PGC1α/TFAM and ERα positivity as well as a high Ki-67 index (summarized in Fig. 5). Interestingly, the CCC profile had developed also in an EOC cell line that is not of CCC origin and which had been made highly chemoresistant *in vitro*.

Here, out of the nine tumour samples that were double-negative for both PGC1α/TFAM, eight were CCC. In view of its characteristic histology, accumulation of intracellular glycogen and its highly chemoresistant nature, CCC represents a distinct clinicopathological subtype of requires new treatment strategies [32]. We believe that our findings support this notion; furthermore, our data also provide mechanistic insights into where this chemoresistance comes from.

Very little is reported on the role of PGC1α in cancer in general, and even less in chemoresistance. Low levels of PGC1α have been reported in ten EOC samples compared with normal ovaries, and PGC1α overexpression *in vitro* led to upregulation of Bax, downregulation of Bcl-2 and to apoptosis [33]. PGC1α was reported to be low also in 15 out of 17 colon cancer biopsies compared to normal [34]. There were, however, no reported results on any association with data on treatment effects or chemoresistance. Nevertheless, the role of the PGC1α/oestrogen-related receptors (ERR) axis is known to be instrumental in regulation of cancer cell metabolism (reviewed in [35]), which in turn is known to affect responses to chemotherapy [8], [36].

Low expression of PGC1α might be expected to correlate with lower mitochondrial content. Mitochondria are required for the full effect of some chemotherapeutic drugs, e.g. cisplatin, due to their role in apoptosis and a requirement for mitochondrially produced ROS [37]. However, we did not observe any correlation between PGC1α expression and mitochondrial content, based on VDAC and MT-CO2 expression. PGC1α is involved also in regulation of antioxidant defence [21], [38], and the observed low levels of PGC1α would thus be expected to not reduce ROS production and thereby the effect of platinum. Therefore, the role of PGC1α in EOC, particularly in notoriously resistant CCC [32], is likely not linked to antioxidant defence.

TFAM is a requirement for normal replication and transcription of mtDNA [17], [18]; this is evidenced as reduced mtDNA content in colon carcinoma cell lines as well as in biopsies carrying truncating mutations in TFAM [39]. Here, of the four samples that did not express mitochondrially encoded MT-CO2, three did not show detectable TFAM. This suggests the possibility that a detectable proportion of EOC tumours do not have functional respiration, as MT-CO2 provides the substrate-binding site of Complex IV in the respiratory chain [40]. Based on the literature [18], [41], we hypothesised that minute amounts of TFAM are sufficient for transcriptional activity and that the undetectability of TFAM protein might be due to Lon-mediated degradation [19], [20]. However, as there was no significant correlation between the expression of TFAM and Lon, this was not the case.

CCC generally shows low Ki-67 index [32]. A correlation between TFAM and Ki-67 has, to our knowledge, not been studied. However, in analogy with the report that in highly proliferative transformed lymphoblastoid cells, increased expression of TFAM was suggested to be associated with protection from oxidative stress induced damage [42], we speculate that highly proliferative HGSC tumours, as indicated by Ki-67, upregulate TFAM as part of an antioxidant defence. Our in vitro results are also in accordance with this, since SKOV-3-R cells showed downregulation of TFAM and grow more slowly than the parental cells (Fig. S4). Moreover, TFAM expression correlated positively with ERα, which might reflect a potential for growth as well as for an efficient oxidative phenotype. In line with previous studies [26], [43], expression of ERα varied across the EOC subtypes, with low levels in CCC.

In a mouse model, SKOV-3 tumour xenografts (non-CCC, EOC cells) treated with trastuzumab were at post-treatment found to be heterogeneous with large areas of ERα negative CCC morphology [44]. This finding is interesting in the present context, as the multiresistant SKOV-3-R cells had acquired the CCC profile, compared to parental SKOV-3 cells. Furthermore, SKOV-3-R showed accumulation of glycogen, again in accordance with a CCC phenotype. Knocking down *PPARGC1A* mRNA using siRNA in the SKOV-3 cells down-regulated PGC1α mRNA as well as protein levels seen at 72 h post transfection (Figs. S5A–B). TFAM protein was also reduced, although *TFAM* mRNA expression was restored at this time point (Figs. S5A–B). Although not significant, the knockdown cells showed a clear tendency of reduced response to cisplatin treatment compared to the negative control cells (Fig. S5C). This tendency was not due to reduced growth, as knocking down *PPARGC1A* did not alter the growth rate or the expression of Ki-67 positive cells. We speculate that long-term downregulation of PGC1α and/or TFAM is necessary to decrease growth rate over time.

Although our SKOV-3 cell line model represents only itself, we suggest that downregulation of PGC1α activity as part of a chemoresistant phenotype profile might not be restricted to ovarian CCC. Low PGC1α activity would be expected in tumour contexts where loss of LKB1/AMPK represents loss of tumour suppressor function [45]. In keeping with a context-dependent tumour suppressor and oncogenic role for AMPK [45], the comparatively high levels of PGC1α expression that we observed in the highly proliferative, i.e., with high Ki-67 expression, HGSC tumours could then be suggested to belong to a context of AMPK-mediated protection from proliferation-related oxidative stress.

In summary, we propose that the profile consisting of PGC1α/TFAM downregulation and decreased ERα/Ki-67, and likely also glycogen accumulation, is representative of CCC and chemoresistance. We are currently investigating an underlying mechanistic explanation for this association. Fig. 5 summarises how HGSC and CCC may represent opposites with regard to the profile. Altogether, the findings corroborate the need to take into account the diversity in EOC and to develop specific treatment strategies for each subtype.

## Supporting Information

Figure S1 **Expression of Ki-67, MT-CO2 and Lon in EOC.** Representative negative and positive immunohistochemical staining of Ki-67, MT-CO2 and Lon in EOC (magnification: 400x, scale bar shows 500 µm).
(TIF)

Figure S2 **Ki-67 index distribution in different EOC subtypes.** Distribution of Ki-67 index varied significantly across the different EOC subtypes (Mann-Whitney U, *p* = 0.003). High-grade serous carcinoma (HGSC) (*n* = 21), clear cell (CC) (*n* = 14), endometrial carcinoma (EC) (*n* = 10), mucinous carcinoma (MC) (*n* = 7) and low-grade serous carcinoma (LGSC) (*n* = 1).
(TIF)

Figure S3 **Ki-67 index distribution in EOC tumours depending on expression of PGC1α and TFAM.** Distribution of Ki-67 index varied significantly across the different groups of tumours with PGC1α-/TFAM- (*n* = 9), PGC1α-/TFAM+ (*n* = 2), PGC1α+/TFAM- (*n* = 9) and PGC1α+/TFAM+ (*n* = 33) (Kruskal-Wallis test, *p* = 0.048).
(TIF)

Figure S4 **Growth rates in EOC SKOV-3 cells and the multiresistant subline SKOV-3-R.** Growth rates in SKOV-3 and SKOV-3-R cells assessed as cellular protein at given time points using the sulphorhodamine B assay (*n* = 4). Data are expressed as fold increase from *t* = 0 h. S.E.M. error bars were too small to be visualized, except where shown.
(TIF)

Figure S5 **PGC1α/TFAM expression and response to cisplatin treatment in SKOV-3 PPARGC1A siRNA knockdown cells.** SKOV-3 cells were treated with PPARGC1A siRNA knockdown or siRNA negative control; (A) gene expression of *PPARGC1A* and *TFAM* was evaluated at 72 h post-transfection by qRT-PCR (*n* = 3). Expression levels were normalised to *ACTB*. Error bars represent S.E.M. (B) representative western blot showing protein expression at 72 h post-transfection of PGC1α, Ki-67 and TFAM. β-tubulin was used as loading control. (C) At 24 h post-transfection, SKOV-3 PPARGC1A siRNA knockdown and siRNA negative control cells were treated with indicated doses of cisplatin for 48 h, whereafter cellular protein was measured using the sulphorhodamine B assay (*n* = 3). Data are expressed as percent of untreated cells. Error bars represent S.E.M.
(TIF)

File S1 **Supporting tables. Table S1, Expression of MT-CO2 in EOC.** Shown is the distribution of positive and negative expression of MT-CO2 in the total sample, in the five EOC subgroups and in TFAM-expressing and non-expressing samples, respectively. **Table S2, Expression of Lon in EOC.** Shown is the distribution of positive and negative expression of Lon in the total sample, in the five EOC subgroups and in TFAM-expressing and non-expressing samples, respectively.
(DOCX)

Raw Data S1 S_PCR: Gene expression raw data given as Ct, delta-Ct (DCt) and delta-delta-Ct (DDCt) values, respectively, for results shown in Figs. 1 and 4, and Fig. S5. S_Spect: Spectrophotometry raw data given as absorbances and calculations based thereupon, for results shown in Figs. 4 and S5. S_IHC: The finalized scoring of immunohistochemical stainings.
(ZIP)
