# Supplementary figures and images for: Expression of Mitochondrial Regulators PGC1α and TFAM as Putative Markers of Subtype and Chemoresistance in Epithelial Ovarian Carcinoma
Source: PLoS One. 2014 Sep 22;9(9):e107109. doi: 10.1371/journal.pone.0107109 (PMC4170973; doi:10.1371/journal.pone.0107109)

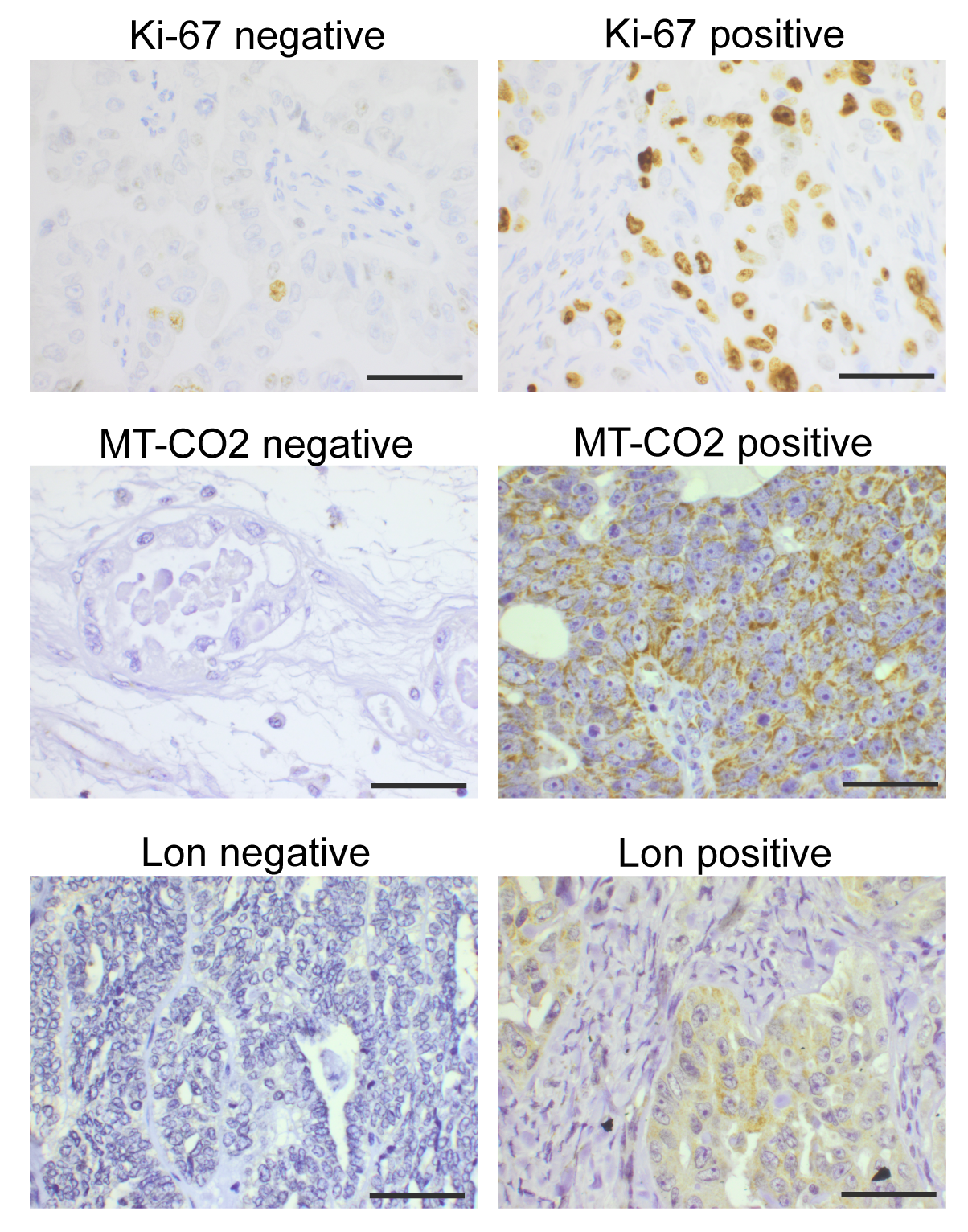

Supplement: Figure S1 — Expression of Ki-67, MT-CO2 and Lon in EOC. Representative negative and positive immunohistochemical staining of Ki-67, MT-CO2 and Lon in EOC (magnification: 400x, scale bar shows 500 µm). (TIF) [file pone.0107109.s001.tif]

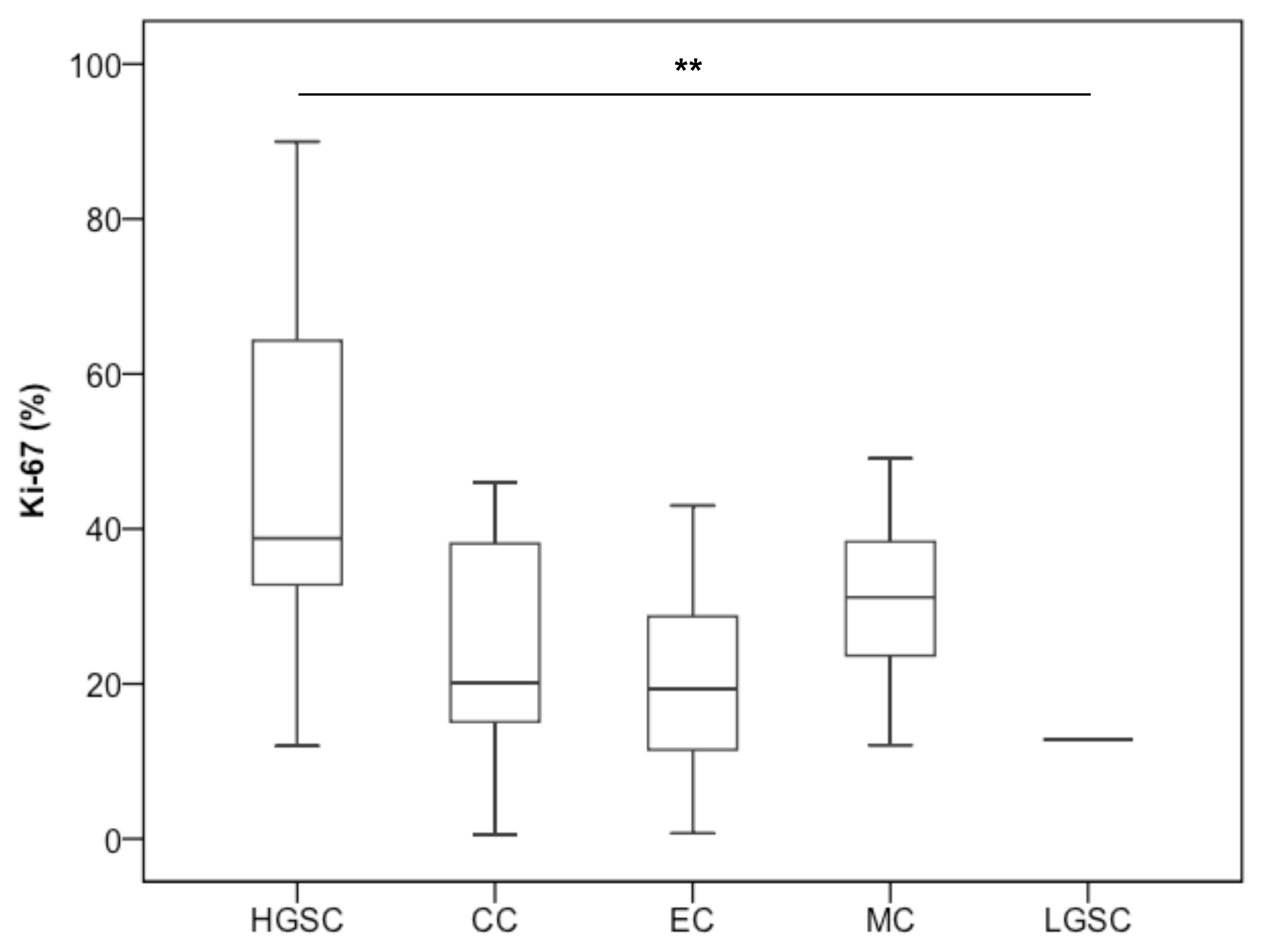

Supplement: Figure S2 — Ki-67 index distribution in different EOC subtypes. Distribution of Ki-67 index varied significantly across the different EOC subtypes (Mann-Whitney U, p = 0.003). High-grade serous carcinoma (HGSC) (n = 21), clear cell (CC) (n = 14), endometrial carcinoma (EC) (n = 10), mucinous carcinoma (MC) (n = 7) and low-grade serous carcinoma (LGSC) (n = 1). (TIF) [file pone.0107109.s002.tif]

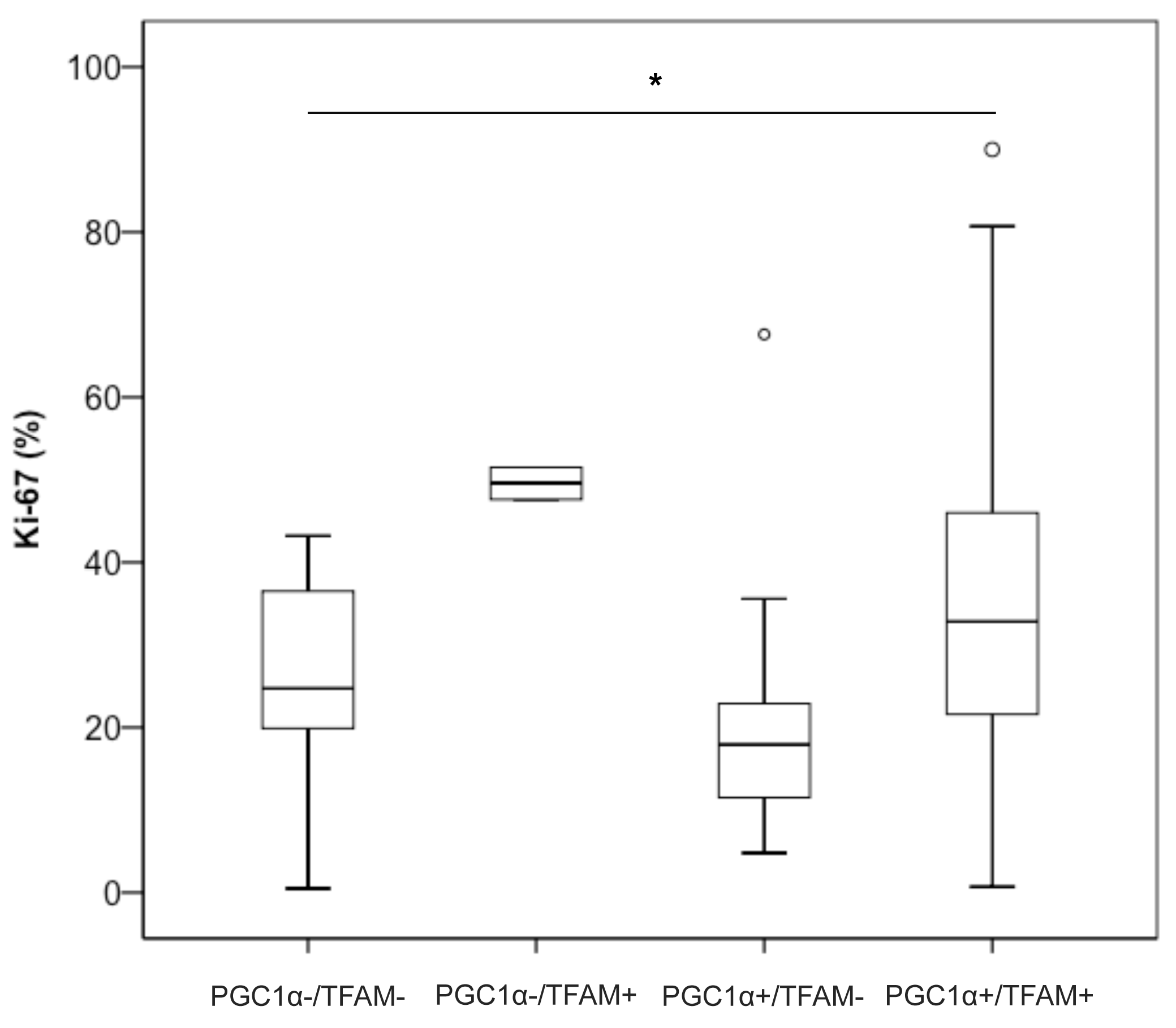

Supplement: Figure S3 — Ki-67 index distribution in EOC tumours depending on expression of PGC1α and TFAM. Distribution of Ki-67 index varied significantly across the different groups of tumours with PGC1α-/TFAM- (n = 9), PGC1α-/TFAM+ (n = 2), PGC1α+/TFAM- (n = 9) and PGC1α+/TFAM+ (n = 33) (Kruskal-Wallis test, p = 0.048). (TIF) [file pone.0107109.s003.tif]

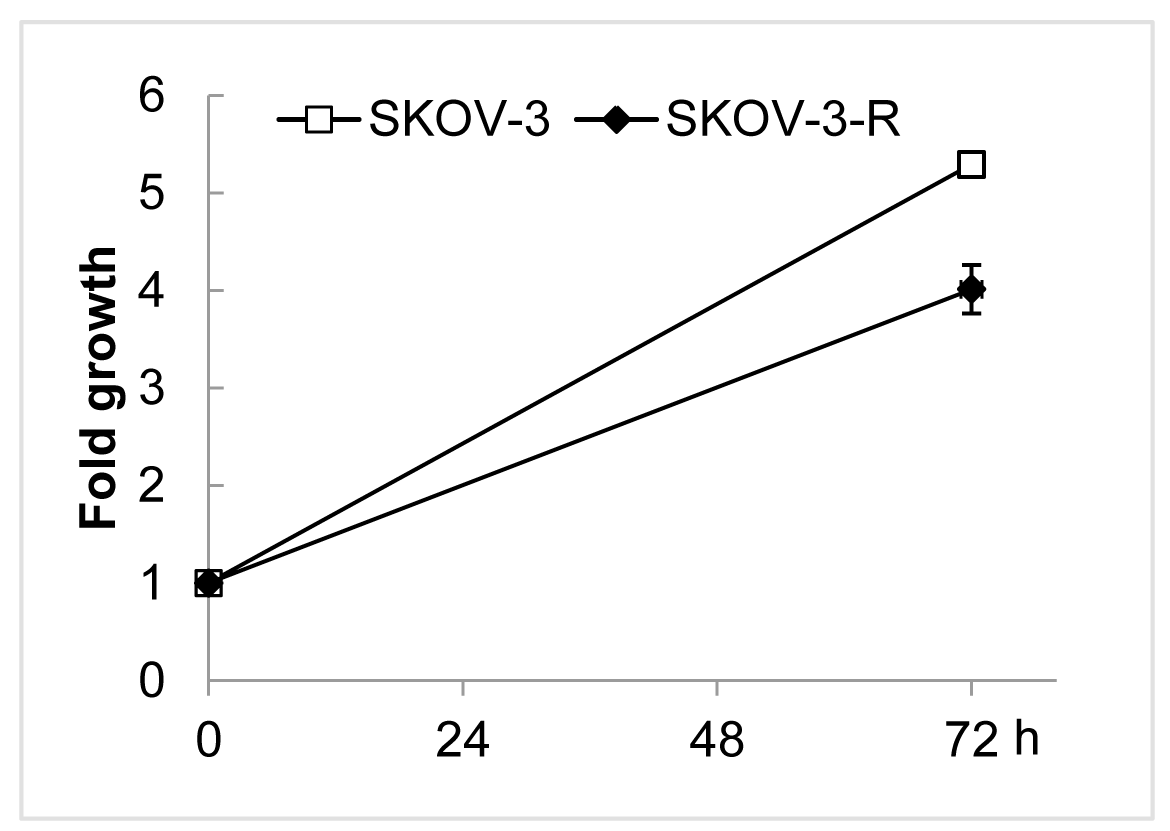

Supplement: Figure S4 — Growth rates in EOC SKOV-3 cells and the multiresistant subline SKOV-3-R. Growth rates in SKOV-3 and SKOV-3-R cells assessed as cellular protein at given time points using the sulphorhodamine B assay (n = 4). Data are expressed as fold increase from t = 0 h. S.E.M. error bars were too small to be visualized, except where shown. (TIF) [file pone.0107109.s004.tif]

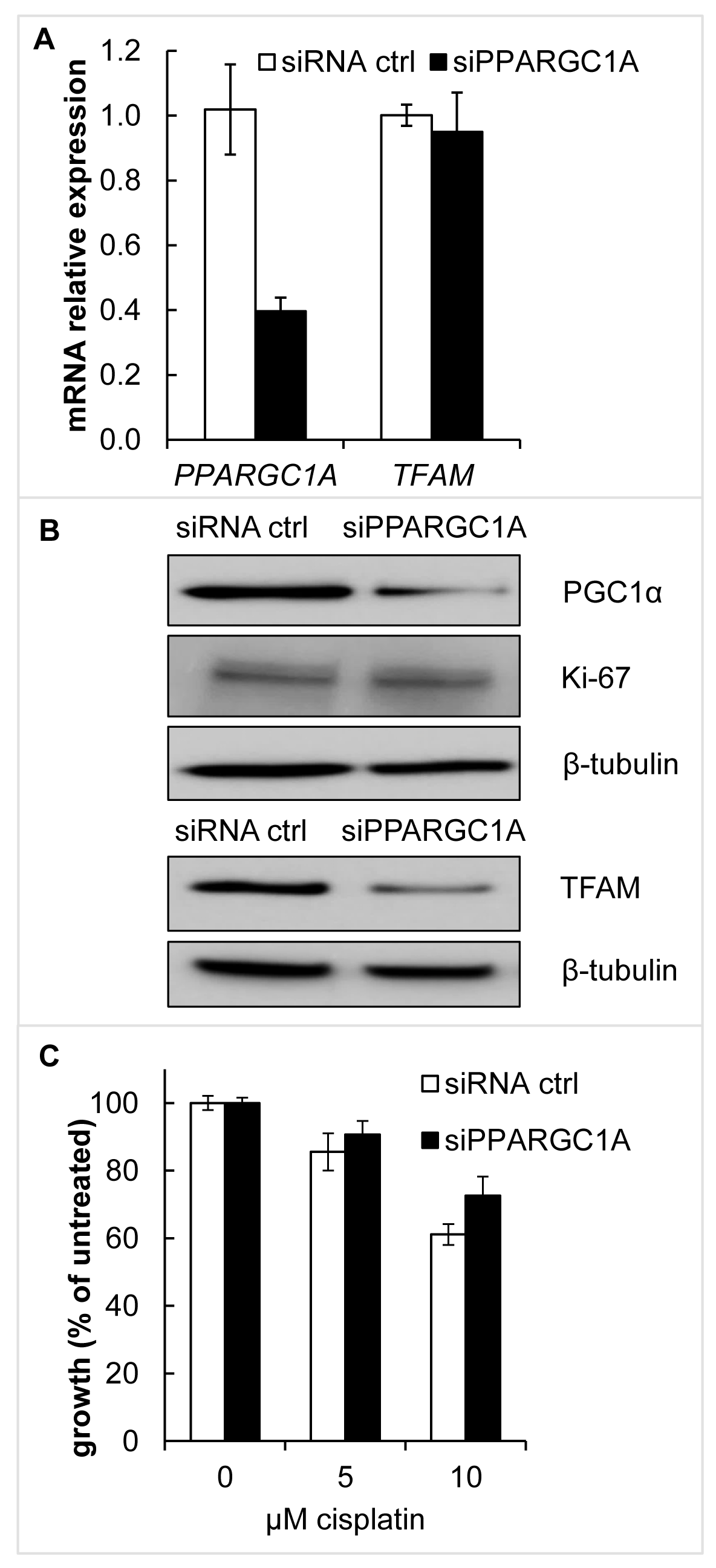

Supplement: Figure S5 — PGC1α/TFAM expression and response to cisplatin treatment in SKOV-3 PPARGC1A siRNA knockdown cells. SKOV-3 cells were treated with PPARGC1A siRNA knockdown or siRNA negative control; (A) gene expression of PPARGC1A and TFAM was evaluated at 72 h post-transfection by qRT-PCR (n = 3). Expression levels were normalised to ACTB. Error bars represent S.E.M. (B) representative western blot showing protein expression at 72 h post-transfection of PGC1α, Ki-67 and TFAM. β-tubulin was used as loading control. (C) At 24 h post-transfection, SKOV-3 PPARGC1A siRNA knockdown and siRNA negative control cells were treated with indicated doses of cisplatin for 48 h, whereafter cellular protein was measured using the sulphorhodamine B assay (n = 3). Data are expressed as percent of untreated cells. Error bars represent S.E.M. (TIF) [file pone.0107109.s005.tif]
