# Supplementary material for: Expression of Mitochondrial Regulators PGC1α and TFAM as Putative Markers of Subtype and Chemoresistance in Epithelial Ovarian Carcinoma
Source: PLoS One. 2014 Sep 22;9(9):e107109. doi: 10.1371/journal.pone.0107109 (PMC4170973; doi:10.1371/journal.pone.0107109)
Supplement: File S1 — Supporting tables. Table S1, Expression of MT-CO2 in EOC. Shown is the distribution of positive and negative expression of MT-CO2 in the total sample, in the five EOC subgroups and in TFAM-expressing and non-expressing samples, respectively. Table S2, Expression of Lon in EOC. Shown is the distribution of positive and negative expression of Lon in the total sample, in the five EOC subgroups and in TFAM-expressing and non-expressing samples, respectively. (DOCX) [file pone.0107109.s006.docx]

**Supporting Information, Tables**

| **Table S1.** **Expression of MT-CO2 in EOC** | | | |
| --- | --- | --- | --- |
| Clinical Variable | MT-CO2 Negative | MT-CO2 Positive | *p-value* |
| Number of Cases, *n* (%) | 4 (8) | 47 (92) |  |
| Subtype |  |  | n.s. |
| High-Grade Serous, *n* (%) | 1 (5) | 19 (95) |  |
| Clear Cell, *n* (%) | 1 (7) | 13 (93) |  |
| Endometrioid, *n* (%) | 2 (20) | 8 (80) |  |
| Mucinous, *n* (%) | 0 (0) | 6 (100) |  |
| Low-Grade Serous, *n* (%) | 0 (0) | 1 (100) |  |
| TFAM |  |  | n.s. |
| Negative, *n* (%) | 3 (17) | 15 (83) |  |
| Positive, *n* (%) | 1 (3) | 32 (97) |  |

Abbreviations: EOC: epithelial ovarian carcinoma; MT-CO2: cytochrome c oxidase subunit 2; n.s.: not significant; TFAM; mitochondrial transcription factor A.

| **Table S2.** **Expression of Lon in EOC** | | | |
| --- | --- | --- | --- |
| Clinical Variable | Lon Negative | Lon Positive | *p-value* |
| Number of Cases, *n* (%) | 10 (21) | 38 (79) |  |
| Subtype |  |  | 0.032 |
| High-Grade Serous, *n* (%) | 1 (5) | 18 (95) |  |
| Clear Cell, *n* (%) | 3 (23) | 10 (77) |  |
| Endometrioid, *n* (%) | 5 (56) | 4 (44) |  |
| Mucinous, *n* (%) | 1 (17) | 5 (83) |  |
| Low-Grade Serous, *n* (%) | 0 (0) | 1 (100) |  |
| TFAM |  |  | n.s. |
| Negative, *n* (%) | 5 (29) | 12 (71) |  |
| Positive, *n* (%) | 5 (16) | 26 (84) |  |

Abbreviations: EOC; epithelial ovarian carcinoma; n.s.: not significant; TFAM: mitochondrial transcription factor A.

**Supplemental Results; figure legends**

**Figure S1. Expression of Ki-67, MT-CO2 and Lon in EOC.** Representative negative and positive immunohistochemical staining of Ki-67, MT-CO2 and Lon in EOC (magnification: 400x, scale bar shows 500 μm).

**Figure S2.** **Ki-67 index distribution in different EOC subtypes.** Distribution of Ki-67 index varied significantly across the different EOC subtypes (Mann-Whitney U, *p* = 0.003). High-grade serous carcinoma (HGSC) (*n* = 21), clear cell (CC) (*n* = 14), endometrial carcinoma (EC) (*n* = 10), mucinous carcinoma (MC) (*n* = 7) and low-grade serous carcinoma (LGSC) (*n* = 1).

**Figure S3.** **Ki-67 index distribution in EOC tumours depending on expression of PGC1α and TFAM.** Distribution of Ki-67 index varied significantly across the different groups of tumours with PGC1α-/TFAM- (*n* = 9), PGC1α-/TFAM+ (*n* = 2), PGC1α+/TFAM- (*n* = 9) and PGC1α+/TFAM+ (*n* = 33) (Kruskal-Wallis test, *p* = 0.048).

**Figure S4. Growth rates in EOC SKOV-3 cells and the multiresistant subline SKOV-3-R.** Growth rates in SKOV-3 and SKOV-3-R cells assessed as cellular protein at given time points using the SRB assay (*n* = 4). Data are expressed as fold increase from *t* = 0h. S.E.M. error bars were too small to be visualized, except where shown.

**Figure S5. PGC1α/ TFAM expression and response to cisplatin treatment in SKOV-3 PPARGC1A siRNA knockdown cells.** SKOV-3 cells were treated with PPARGC1A siRNA knockdown or with siRNA negative control; (A) gene expression of *PPARGC1A* and *TFAM* was evaluated at 72h post-transfection by qRT-PCR (*n* = 3). Expression levels were normalised to *ACTB*. Error bars represent S.E.M. (B) representative western blot showing protein expression at 72h post-transfection of PGC1α, Ki-67 and TFAM. β-tubulin was used as loading control. (C) At 24h post-transfection, SKOV-3 PPARGC1A siRNA knockdown and siRNA negative control cells were treated with indicated doses of cisplatin for 48h, whereafter cellular protein was measured using the SRB assay (*n* = 3). Data are expressed as percent of untreated cells. Error bars represent S.E.M.
